# Analysis of institutional daily domestic water consumption dynamics due to COVID-19 pandemic, a case study of Adama Science and Technology University

**DOI:** 10.1007/s13201-022-01637-z

**Published:** 2022-04-15

**Authors:** Segni Lemessa Tesgera, Sissay Dechasa Hailemariam, Gemechis Guta Tucho

**Affiliations:** Department of Water Resources Engineering, Institute of Water Resources and Irrigation Engineering,Center of Excellence, Adama Science and Technology University, 1888, Adama, Ethiopia

**Keywords:** COVID-19, Hand wash, Daily domestic water consumption, Institutions, Water supply

## Abstract

The coronavirus pandemic outbreak is constantly changing the way of people’s life. To minimize or stop the transmission of the virus, several different measures have been taken by countries worldwide and including in Ethiopia. The World Health Organization (WHO), the Ethiopian Ministry of Health, and the Ethiopian Public Health Institute recommended frequent hand wash with soap and water as one of the prevention measures for the transmission of the COVID-19 pandemic. Conversely, the provision of water supply, sanitation, and hygiene is the main problem in developing countries. After the COVID-19 outbreak, the water related challenges exacerbated the situation before the pandemic existed. Accordingly, the additional supply of water is required to sufficiently provide water for hand wash. This paper mainly addresses how much additional water is required daily for domestic consumption after the coronavirus pandemic by educational institutions, Adama Science and Technology University as a case study. The data was collected with a questionnaire by the randomly selected respondents and analyzed by SPSS (Statistical package for social sciences).The analysis shown that the consumption of water has been changed after the Corona virus (COVID-19) pandemic existed. The highest percentage of the responses to the questionnaire, more than 35% require water (2–5) liters per day and more than 60% of the respondents need additional water up to 5 L per day after the pandemic existed. Based on the analysis, total daily water use by the institute before the COVID-19 existed was 24.2 m^3^/day. These amounts of water not sufficiently satisfy the daily water requirements of the institution even before the pandemic. After the pandemic existed, the water consumption drastically increased to 29 m^3^/day. Furthermore, the study recommends the provision of additional water supply sources or water demand management to reduce the impact of the pandemic on water availability.

## Introduction

In late 2019, a severe respiratory disease emerged, known as novel coronavirus disease 2019. The pathogen responsible for COVID-19 is severe acute respiratory syndrome coronavirus 2 (SARS-CoV-2), also stated as the COVID-19 virus (World Health Organization 2020).

The coronavirus pandemic outbreak has become a global problem since March 11, 2020 (WHO).This problem constantly changes the way of living of human beings. Several different measures are taken by countries in the world. The restriction of public movements, school closure, and worldwide programs like conferences, sports activities were canceled (Balacco et al. 2020). Likewise, in Ethiopia, the Ministry of Health and Ethiopian Public Health Institute have been working together in the prevention of the COVID-19 disease and they provide information updates regarding coronavirus. As a prevention measure, frequent hand wash with water and soap is recommended by the Ethiopian Ministry of Health (Ministry of Health and the Ethiopian Public Health Institute (EPHI) 2020).

The provision of proper sanitation, safe water, and hygienic conditions are important in protecting human health during all infectious disease outbreaks, including the COVID-19. Ensuring good and frequently applied water, sanitation, hygiene (WASH) practices and waste management practices in communities, homes, schools, marketplaces, and health care facilities will further help to prevent human-to-human transmission of the COVID-19 virus (WHO 2020). After the COVID-19 pandemic disease has spread all over the world, the significances of regular hand-washing with clean water, sanitation, and wastewater management have been rediscovered. In developing countries availability of clean water has traditionally been a concern and the situation of COVID-19 challenges water supply more than before the pandemic existed (Tortajada 2021). Water availability is decreasing as population growth increases (Dolphin 2007),which affects the provision of water supply. As a result, most families in developing countries have problem of providing available and quality of piped water due to financial and technical capabilities. In semi-arid regions of Ethiopia getting available water is challenging; in Adama city the bore holes are up to 500 m deep and available water is very hot. This water is not frequently used for drinking purposes. In Adama Science and Technology University campus; the university community use packed water for drinking purposes. They can buy the bottled water from small shops, supermarkets and packed water distributors.

Also, COVID-19 pandemic will degrade the trust in water supply even more in the coming years (Tortajada and Biswas 2020). Water is not always available at the right time and place to meet the demands. By the year 2025, approximately 3 billion people could be living in water stress, without meeting their water requirements (United Nations Development Programs 2006). The combination of water stress with the COVID-19 pandemic requires the ultimate solution particularly in developing countries. The trend of per capita water consumption is increasing due to economic development and it is better to identify the major factors influencing the water demand (Bradley 2004). Based on the recent study, Preventing the coronavirus pandemic likely increases water demand for domestic and health care issues (Cooper 2020). Daily water demand required by institutions changed as hand wash is considered a prevention measure. Strengthening water security is important for preventing the coronavirus pandemic. The factors that affect water use are monthly income, time spent to get water, household size, and capacity of the storage tank (Nnaji et al. 2013).

As an alternative measures, several suggestions are proposed in short term and long term strategies.

The ultimate solution was suggested by (Haddout et al. 2020) as a short-term and long-term strategy. The short-term strategies were; run the dishwasher and clothes washer only when they are fully loaded, fix leaks: conserves water and saves repair costs, education (public awareness), setting up awareness programs through social media users for sharing information, providing training and capacity building. In the long term, it was suggested to develop water management policies, search for alternate sources of water, and preparation of a management action plan for the wise use of water resources. Water supply provision in Ethiopia determined by several factors and water supply delivered to communities, institutions, schools and health care facilities varies in urban and rural parts of the country. In rural areas getting potable water was challenging due to lack of economic and technical capabilities and in urban areas the water demand is not fully satisfied. To overcome water supply challenges, the Ethiopian government and Non-Governmental Organizations (NGO) now working in rural water supply and expansion of existing water supply system in urban areas. In Ethiopia the sources of water are either surface water or groundwater. Surface water is mostly contaminated and it is not used for domestic use without treatment. Conversely, ground water quality is safe, but most boreholes are deep and it needs huge amount of energy to deliver water. The urban population in Ethiopia recently increasing when compared to 1990 and early 2020s. As a result, the water supply provided is not enough for satisfying domestic and public water demand.

Water consumption in an institution is expressed by several types of demand, including domestic, commercial, public and industrial uses. Domestic water consumption includes water for cooking, washing, drinking laundering etc. The public water demand includes water for fire protection, street cleaning, and use in schools and other public buildings. Commercial and industrial water requirements include water for offices, hotels, laundries, restaurants, stores and manufacturing companies. There is usually a wide variation in total water demand among different communities. Water availability is the main problem to ensure safe water supply in ASTU after COVID-19 pandemic existed in Ethiopia. Technical capabilities and having weak economy in maintaining and operating of existing water supply system are also the main challenges. As a solution using hand sanitizers could be recommended in case of using water for hand wash. Also, it is expected from government to provide the incentives and approve the budget that will develop the water supply sector. Finally, the water supply sector could work on water demand management system and creating awareness in a community how to use water efficiently and sustainably.

### The study area

**Fig. 1:**
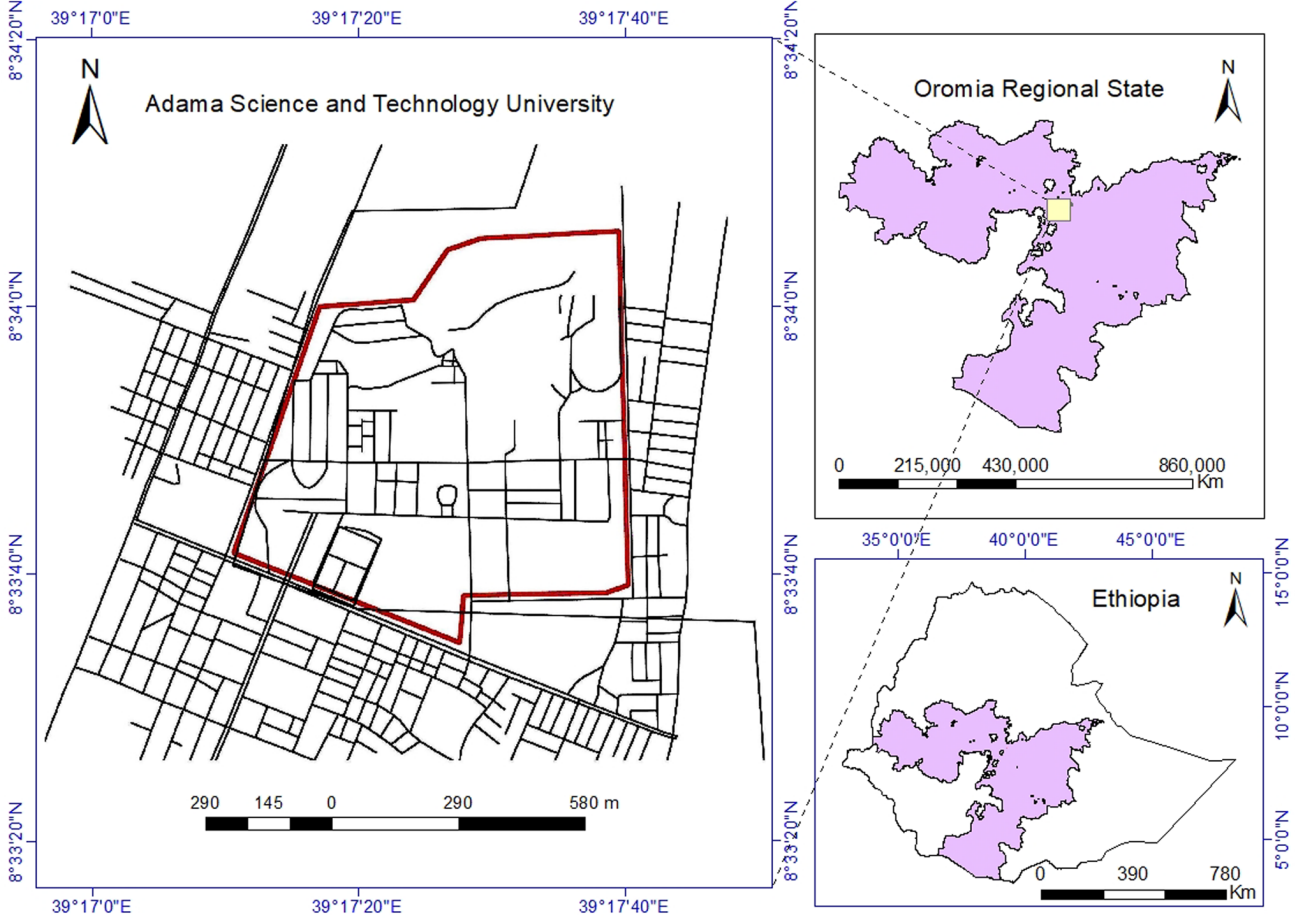
Location map of the study area

Adama Science and Technology University (ASTU) is located in Adama town which is found in the Eastern part of Addis Ababa, the capital city of Ethiopia. It is laid between 8°33′20′′–8°34′20′′ N latitudes and 39°17′00′′–39°40′00′′ E longitudes (Fig. 1) with an altitude of 1660 m above mean sea level. This University is one of the emerging Ethiopian Public Universities located in Oromia regional state. ASTU aspires to be the first choice in Ethiopia and the premier center of excellence in applied science and technology in Africa by 2030 (Senate legislation 2017).

### Object of the study

The specific objectives of the study were to quantify the institutional water consumption at Adama Science and Technology University (ASTU) and to analyze the daily dynamics of domestic water consumption before and after the coronavirus pandemic at educational institutions. The institute, ASTU uses ground water as sources of water supply. There are six ground water bore holes. Four bore holes are now functional in supplying water. The ground water is pumped to the elevated reservoirs. The pumps are operated for 8 h and deliver water to the reservoirs. Finally water is supplied to users from the reservoirs in the morning from 6:00 AM to 10:00 AM Ethiopian local time. The supplied water was not continuous which will result less water availability during peak water demand.

## Methodology

The study was conducted by data from questionnaires and field survey. First, preliminary data collection conducted, then preparation and the collection of the questionnaire, and the field survey. The information collected at the preliminary stage arranged the fundamental parameters recommended in sampling procedure. The parameters are daily per capita water consumption (drinking, cooking, washing, bathing, and toilet), water availability, and water quality for drinking purposes, additional institutional water consumption after COVID-19, and total daily water consumption by the educational institutions. In the statistical representation simple random sampling used to obtain data for the overall analysis of the parameters. The daily water requirement is determined by the socio-economic status of the region; which is mainly characterized as metropolitan, urban, rural, rich, poor, food habits level and type of developments, sewerage system, and public water supply. Daily water requirement (*Q*_*d*_) is calculated based on the per capita water use. First the per capita water consumption (*C*) for different water users (students, academic and administrative staff) were estimated with sample data collected.

The per capita of water consumption of ASTU was represented by average value (demand of all water users), and then total daily water demand was estimated for the selected sample size that represents the whole population in the institution.

The daily water consumption is estimated by the following equations below.

1 $$\user2{ Q}_{{\varvec{c}}} = \user2{C*P}$$

2 $${\varvec{Q}}_{{{\mathbf{day}}\,\,{\mathbf{max}}}} = {\mathbf{1.8}}\user2{* Q}_{{\varvec{c}}}$$

where *C* = Per capita water consumption, *P* = Population, *Q*_*c*_ = Average daily water consumption, and *Q*_day max_ = Maximum daily water consumption.

The research study analyzes the daily water demand (*Q*_*d*_) in two different scenarios. Scenario 1 (S1) considers the water consumption before the Covid-19 pandemic existed and scenario 2 (S2) involves the influence of the Coronavirus pandemic on the water use in ASTU. The questionaries' had been proposed to analyze the water use system in the university, to have insight whether the water supply is sufficient for the requested water demand. Additionally, water availability and quality in the campus was analyzed after data collection. The quality of water inside campus is assessed and discussed whether they use piped water or bottled water for drinking purposes.

### Water demand analysis

The raw data for institutional water demand data was collected as a questionnaire and field visits. The questionnaire data addresses the habit of water changed after coronavirus existed, how much additional liters of water required per day and availability of sufficient water. In field visit the sources of water, water supply distribution system and COVID-19 prevention measures taken by the university were observed. Then the data was analyzed with two scenarios; before and after the coronavirus pandemic existed. Whenever the water supply is greater than the demand required, no water shortage occurred. In contrast, while the domestic demand is greater the water supply water shortage could be expected. The institutional water demand (capita per person per day) was estimated by water consumption of users (students, campus resident staff, nonresident staff, and administrative staff).The daily water consumption is sampled randomly that represent the different water users. The volume of water required is calculated by multiplying the per capita per person by the total number of water users. Finally, maximum daily consumption (Qday max) was estimated to know the maximum daily water requirements. The institutional water consumption analysis was considered for both scenarios between the periods of March, 2019 and December 2020. The scenario 1(S1) time series from the period March, 2019 to December, 2019 and scenario 2 (S2) covers the time period from March 11, 2020, to December, 2020.

### School closure and reopening

School closure and reopening had an impact on the daily water consumption of higher education institutions. From March 16, 2020 to October 2, 2020, Adama Science and Technology University was closed due to restrictions imposed by the Ethiopian government. As a result of the pandemic, 5499 students were out of school. After October 2, 2020, graduating students returned to the university with Coronavirus precautions.

**Fig. 2:**
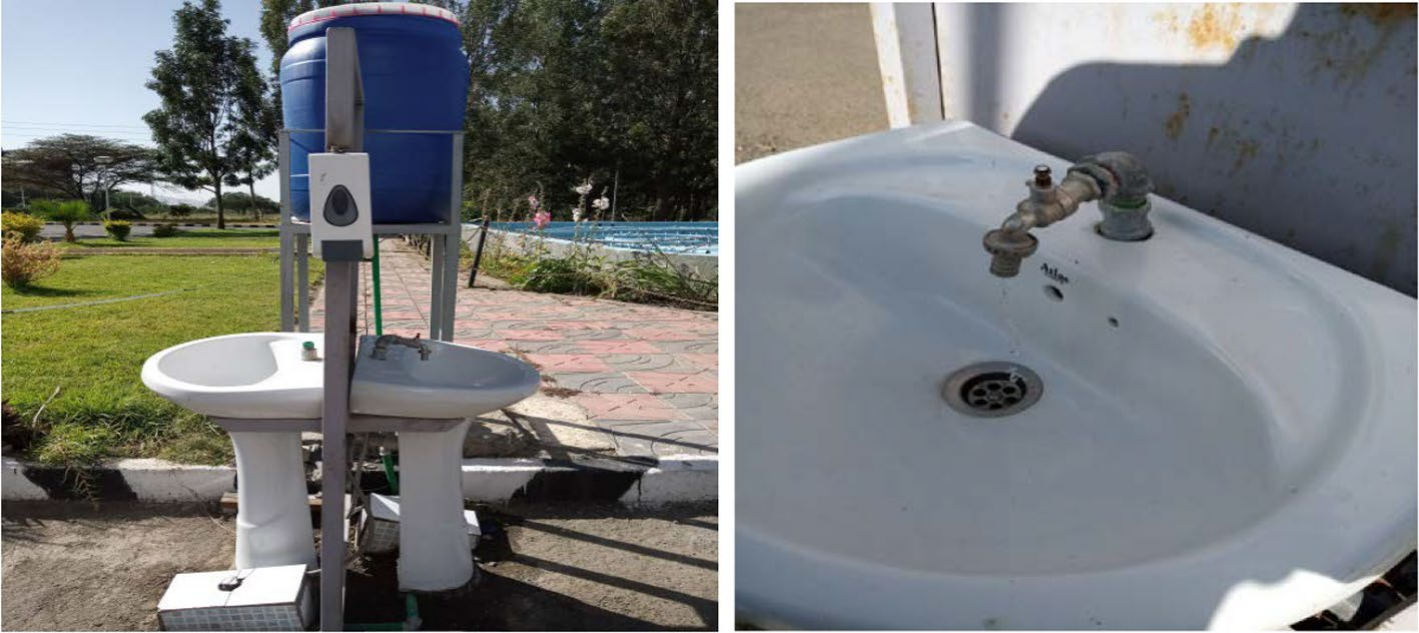
Elevated hand wash constructed inside ASTU campus after COVID-19 existed

After the pandemic to prevent the transmission of coronavirus the Adama Science and Technology University installed and constructed a hand wash in front of the university gate, administrative offices, colleges, and departments (Fig. 2).

### Statistical data analysis

Sample size determination is important in the careful collection of data that often provide better information. When the sample data to be collected is too big it might waste money and time, whereas too small a sample size will not allow revealing reliable results. In order to know the sample size, estimation of confidence level and confidence interval is important. The data analysis for sample size estimated with (95–99%) confidence level and (5–10%), confidence interval. The collected sample size considered to represent the total population and estimated by the following formula.

3 $${\mathbf{Ss}} = \frac{{{\varvec{Z}}^{2} \user2{*}\left( {\varvec{P}} \right)\user2{*}\left( {1 - {\varvec{P}}} \right)}}{{{\varvec{C}}^{2} }}$$

where Ss = sample size, *Z* = *Z* value, *P* = percentage picking a choice, *c* = confidence interval.

**Fig. 3:**
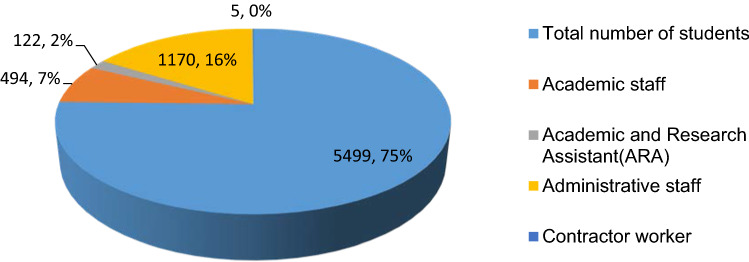
Number of water users in Adama science and Technology University campus

The sample size was estimated by readily available software. The software is available alternatively online at: (https://www.surveysystem.com/sscalc.htm). Totally 95 number of samples (students, academic and research and administrative staff) was collected randomly to represent the whole population of 7885 with 95% confidence level and 10% confidence interval. In Adama Science and Technology University the total number of students’ population is 5499 with 75%, and the number administrative staff 1170 with 16%. The ASTU campus number of academic staff is 494 and academic and research assistant is 122. The detail of number population is depicted in following Fig. 3.

The students in the campus are considered as resident and they live in dormitory. The population both academic and administrative staffs are composed of resident and non-resident. Out of 494 of academic staff in ASTU campus, 68 of them live in the campus. Likewise, few number of administrative also resides inside the campus (18 households from the total of 1762 (Table 1). Those resident students and staff use more than nonresident staff due to several reasons. Resident staffs need additional water for cooking, washing clothes and bathing.

**Table 1:** Residents and nonresident population in Adama Science and Technology University

Population	Resident	Non resident
Students	5499	0
Administrative	18	1762
Academic	68	426
Total	5585	2188

## Results and discussion

**Fig. 4:**
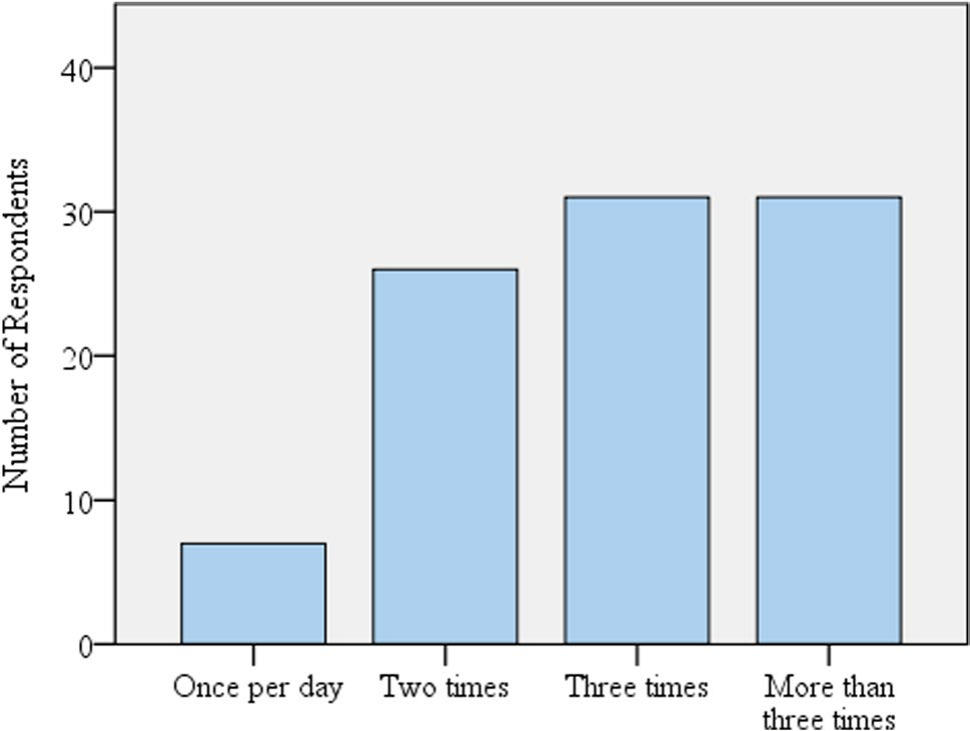
Daily frequency of water consumption and the percentage proportion value inside the ASTU campus before the corona virus pandemic

The study reveals about daily frequency of water consumption, how many liters of water used in consumption as domestic water demand, habit of water use changed before and after COVID-19 pandemic. Additionally, water quality aspect was analyzed whether using piped or bottled packed water used for drinking water. In order to know the water consumption dynamics, the respondents are classified as campus residents and nonresidents. Finally, institutional daily water consumption estimated by the multiplication of per capita consumption with total number of populations (water users). The following bar graph (Fig. 4) depicts the frequency (how many times) water consumed per day (drinking, hand wash, washing clothes, toilets, cooking and baths), inside the university before the Covid-19 existed. The respondents mostly use water three times and more than three times with a total of more than 65% and small portion of respondents’, less than (7%) use water once per day. How many times the respondents, need water in one day was represented in average value expressed by (frequency and its percentage) presented in (Fig. 4).

Before the COVID-19 pandemic existed, out the total sample collected, shown in (Table 2), 20% of respondents need water less than 2 L, 37.9% of respondents need water between (3 and 5) liters and 17.9% of respondents need between (10 and 25) liters, and out of the total sample size, only 9.5% of the respondents consume water more than 25 L per day.

**Table 2:** Daily domestic water requirements inside the campus by water users (in liters) before COVID-19 Pandemic existed

The water requirement in ASTU per day				
How much water required (liters) per day?	Frequency	Percent	Valid percent	Cumulative percent
< 2 liters	19	20.0	20.0	20.0
3–5 liters	36	37.9	37.9	57.9
5–10 liters	14	14.7	14.7	72.6
10–25 liters	17	17.9	17.9	90.5
> 25 liters	9	9.5	9.5	100.0
Total	95	100.0	100.0	

Based on the data analyzed, more than half of total population consume water less than 5 L. In Table 2 value of percent, which is shown in 3rd column of table, valid percent, which is shown in the 4th column is similar, the reason is that all respondents replied the questions without missing data. Missing data exists when the respondents have no comment for the question. Comparatively, the campus resident staff and students demand more water than non-resident administrative and academic staff. The reason was that campus resident (staff and students) they use water for different additional purposes such as washing clothes and cooking. Inside the ASTU campus, there are 3 private and 4 students cafeteria. On average the private cafeterias use 200 L of water per day. Also, students consume 1000 L per day in students’ cafeteria for cooking. Based on this water consumption analysis the total daily water required for cooking in ASTU was 4.2 m^3^/day.

**Fig. 5:**
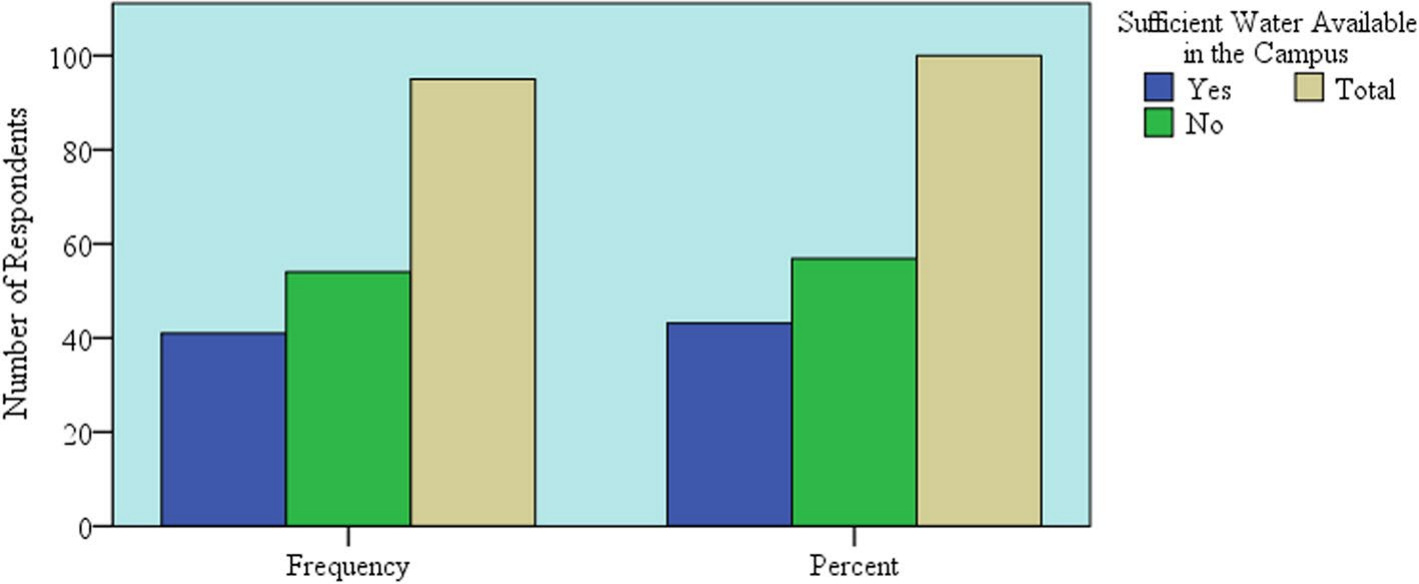
The availability of sufficient water by respondents’ response in ASTU

The bar graph (Fig. 5) illustrates the frequency and percentages of respondents' reply “Yes” or “No” for sufficient available water in the campus. Also, the bar graph shows that 56% of the individuals in the study sample replied the available water supply is not sufficient. The reason was no continuous supply of water for 24 h in ASTU campus. In the analysis (Table 3) shows more than 83% of the respondents had been changed the habit of water use, due to the reason that hand wash and sanitation must be applied frequently. The habit of water use was changed due to frequent hand wash that used to prevent COVID-19 transmission. As WHO and Ethiopian Institute of Public Health recommendation, hands should be cleaned with soap and water for at least 20 s, before and after eating, and after using the restroom. This frequent use of water for hand wash to prevent COVID-19 changes the habit of water use in the campus. As a result, the habit of water use changed after COVID-19 pandemic due to additional water requirement for hand wash and personal hygiene.

**Table 3:** The habit of water use changed after the COVID-19 pandemic

Was the habit of water use is changed after COVID-19?					
		Frequency	Percent	Valid percent	Cumulative percent
Valid	Yes	79	83.2	83.2	83.2
	No	16	16.8	16.8	100.0
	Total	95	100.0	100.0	

When the habit of water use is changed due to COVID-19 pandemic problem, additional water is required for institutional domestic consumption. The respondents who replied “yes” for the habit of water use changed after COVID-19 suggested additional water they need. The following (Table 4) illustrates how many liters of additional water is required in ASTU after the corona virus pandemic existed. The highest percentage of the respondents to the questionnaire, 36.7% require water 2–5 L. More than 60% of the respondents need additional water up to 5 L after the pandemic existed. If additional 10 L of water supply is provided, per person daily as a supply to the institute, more than 84% of the daily water demand could be satisfied.

**Table 4:** Additional water consumption existed in ASTU campus (in liters) after the COVID-19 pandemic

How many liters of additional water are required?				
	Frequency	Percent	Valid percent	Cumulative percent
0–2 liters	20	21.1	25.3	25.3
2–5 liters	29	30.5	36.7	62.0
5–10 liters	18	18.9	22.8	84.8
Above 10 liters	12	12.6	15.2	100.0
Total respondent (answered)	79	83.2	100.0	
No answer	16	16.8		
Total	95	100.0		

**Fig. 6:**
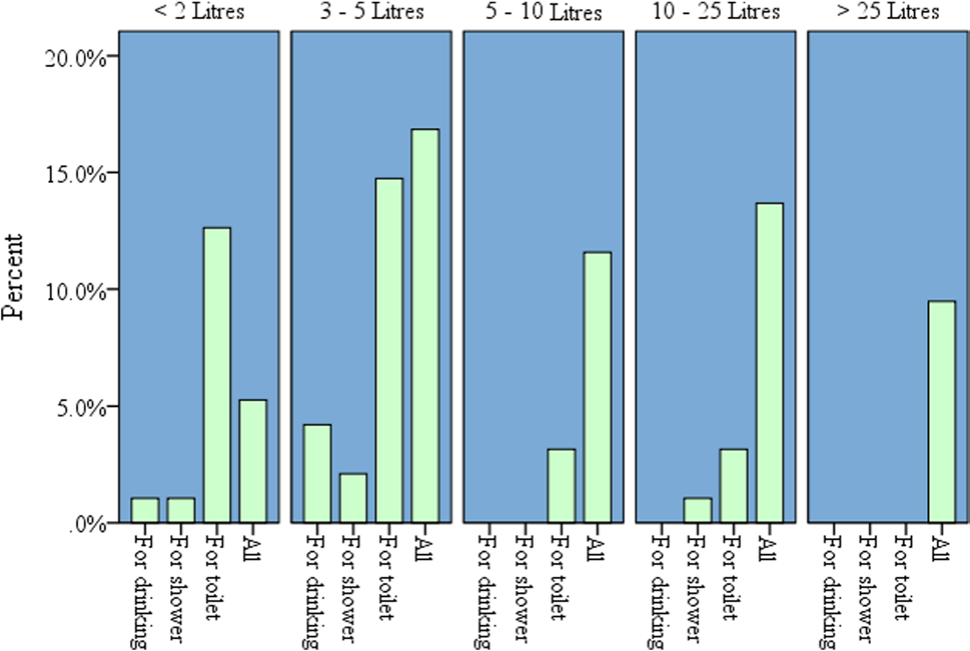
The water required (in liters) for different purposes in percentage before the COVID-19 pandemic existed

The institutional water consumption for different purposes (drinking, bathing, hand wash, toilet) was categorized in liters of water required by the respondents in (Fig. 6). The respondents were replied to how many liters of water they need, which was classified as (less than 2 L, between (3 and 5) liters, (5 and 10) liters, (10 and 25) liters and greater than 25 L.

The analysis shown, the respondents who replied less than 2 L and between (3 and 5) liters of water, they demand water for toilet with the largest proportion. The water consumed for toilet (less than 2 L) and (3–5) liters is greater than 10% while compared to other purposes (drinking and the hand wash), which was less than 5%. Also, the bar graph (Fig. 6) shows the water users’ consumption of water greater than 25 L when they require water for all purposes, otherwise, less than 25 L of water is sufficient for either drinking, toilet or hand wash. Based on the analysis, Adama Science and Technology University institutional daily domestic water demand was in average between (5 and 10) liters.

**Fig. 7:**
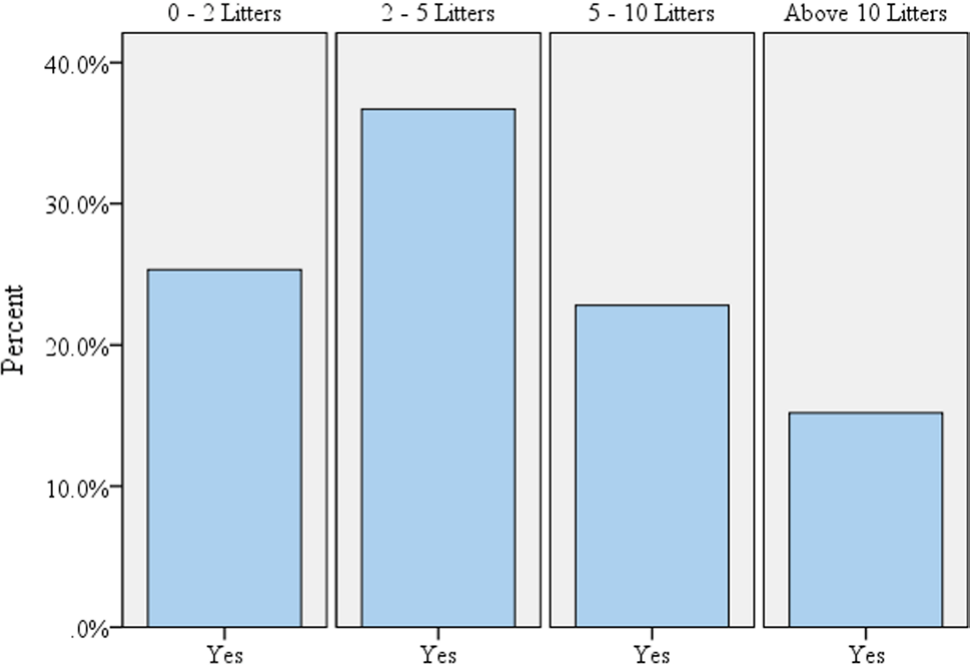
Additional water required in percentage after the COVID-19 pandemic

After the COVID-19 pandemic existed, from the total of 95 respondents, only 79 respondents replied that the habit of water use changed and 16 of them answered habit of water use was not changed after the pandemic. Those respondents who answered yes to the habit of water use changed after the coronavirus pandemic is depicted in the following (Fig. 7). Most of the respondents proposed additional daily water required between (2 and 5) liters, which is more than 35% and more than 60% of respondents require water from (0 to 5) liters. According to the analysis, the institutional daily domestic water was changed due to the COVID-19 pandemic as hand wash and hygiene have a significant change from the previous water consumption.

ASTU campus community use both ground water and the piped water through Adama city water supply enterprise. More than 75% of water consumption is covered from boreholes that owned by ASTU and the other 25% of water withdrawn from Adama city water supply system. The delivered water was safe for drinking, washing dishes and clothes, hand washing and bathing. But the ground water is very hot and not safe for drinking purposes. Academic and administrative staffs use packed bottled water for drinking (68%) and the other (32%) use piped water for drinking purposes (Table 5). Conversely, students in the campus use piped water (61%) and whereas (39%) use packed bottled water. The reason for why such a different number existed was due to economy. Comparatively, students cannot afford money to buy bottled packed water which is currently 10 Ethiopian birr (ETB) for 1L of water.

**Table 5:** Drinking water sources in ASTU based on drinking water consumption

Drinking water sources	Students (%)	Academic and administrative staff (%)
Piped water use	61	32
Packed bottled water use	39	68

The institutional daily water consumption was estimated by the mean value of the respondents' reply. In the case of scenario 1 (before the COVID-19 pandemic) the daily per capita water demand required was 2.59 L and in latter case (after the COVID-19 pandemic), 4 L was requested by the water users (Table 6).

**Table 6:** Daily mean per capita consumption based on the respondents’ water consumption

Case processing summary						
Cases						
Included		Excluded		Total		
*N*	Percent (%)	*N*	Percent (%)	*N*	Percent (%)	Mean
*The water consumption in ASTU per day*						
95	100.0	0	0.0	95	100.0	2.59
*How many liters of additional water is required*						
79	83.2	16	16.8	95	100.0	1.2

Based on the analysis from the respondents total daily water use before the COVID-19 existed was 24.2 m^3^/day. After the pandemic existed, the water consumption drastically changed to 29 m^3^/day. The reason for the change was that the water users (staff, students) responded they demand more water than the supplied even before the pandemic existed. The most water users in the campus are students (5499) who demand domestic water (drinking washing clothes, toilets and hand wash) than others (Non campus resident academic and administrative staff). Non-resident academic and administrative staff (1770) only uses water only for hand wash and toilets in the campus. The analysis of results show low water consumption for non-resident staffs, with low magnitude of daily water consumption and water requirements at ASTU.

## Conclusion

The provision of proper sanitation, safe water, and hygienic conditions are important in human health during all infectious diseases outbreak including Covid-19. The study addresses the additional daily water requirements by the higher education institutions, specifically at Adama Science and Technology University. After the coronavirus (COVID-19) pandemic the habit of water use has been changed. The higher education institutions, particularly Adama Science and Technology University have taken measures to minimize/stop the spread of the coronavirus by implementing hand wash in front of gates and offices. As a result, the provision of a water supply is essential for the proper functioning of the installed hand wash. This situation changes the daily water requirements of domestic water users. Based on the analysis the ASTU community consumes water (5–10) liters per person that could satisfy the need of 70% of the domestic water users. The highest percentage of the respondents to the questionnaire, more than 35% require water (2–5) liters per day. The most water users in the institution are students who demands domestic water (drinking washing clothes, toilets and hand wash) than others (Non campus resident Academic and administrative staff). ASTU campus community use both ground water provided by the University and piped water delivered by Adama city municipality. More than 75% of water consumption is covered by ground water and the other 25% of water withdrawn from Adama city piped water system. The delivered water was safe for drinking, washing dishes, and clothes, hand washing and bathing. But the ground water is very hot and not safe for drinking purposes. Academic and administrative staffs use packed bottled water for drinking 68% and 32% use piped water for drinking purposes. Conversely, students use piped water 61% and whereas 39% use packed bottled water. The reason why such different number (packed bottled water) existed was due to economy. Mostly, the students cannot afford money to buy this bottled packed water. Furthermore, the study recommends the provision of additional water supply sources or water demand management to reduce the impact of the pandemic on water availability.
